# The Poor-Law Service and the War

**Published:** 1914-09-05

**Authors:** 


### THE POOR-LAW SERVICE AND THE WAR.

### The Official Memorandum and Local Effort.

The Local Government Board have issued a memorandum to the Poor-Law Guardians, amongst other local authorities, on the principles which should guide them in their treatment of members of their service who have been called out or volunteer for the war. In this memorandum the Board state that public authorities may properly grant leave of absence to officers in their employ who are already in His Majesty's Forces or who join them with their permission, and may properly make such allowances in respect of salary as they think reasonable in the particular circumstances. The Board consider that local authorities may properly pay temporary substitutes for such officers, and draw attention to the fact that the Treasury Regulations dealing with the case of Civil Servants on naval or military service provide for the payment to them or their representatives during their absence from civil duty of their full pay, less a deduction on account of Navy or Army pay and allowances. Their posts will not be permanently filled during their absence on active service, and that service will count for pension and for increments of salary.

We are glad to be able to record that the Metropolitan Asylums Board and many Boards of Guardians have adopted resolutions embodying the spirit of this memorandum. If it should happen that any Board of Guardians deals with its officers in any less liberal spirit, the fullest publicity should be given to its decisions, so that the force of public opinion may be brought to bear upon it to make it deal with its officers in the spirit in which the Local Government Board consider they should be dealt with.

### Offers of Poor-Law Infirmaries.

The Chertsey Board of Guardians has offered the use of the whole of its workhouse infirmary for the sick and wounded in the war. The Salford Union has offered 150 beds in the Hope Hospital; the Gloucester Union 100 beds in its new infirmary; Nottingham Union a block of buildings at the Nottingham Workhouse; Sheffield Union 100 beds in the Union Hospital; the Newtown and Llanidloes Union a wing of its infirmary; Caistor Union thirty beds; the Wellingborough Union about sixty beds; the Braintree, Battle, and Whitby Unions twenty beds each; the Madeley Union (Shropshire) ten beds; the Chipping Sodbury Union a portion of its infirmary; and the Totnes Union one of its scattered homes. This bare statement of fact is enough to show how general is the share of the Poor-Law infirmaries in the matter of preparation and offers of accommodation for the wounded.

